# Molecular detection of *Anaplasma bovis* in Holstein cattle in the Republic of Korea

**DOI:** 10.1186/s13028-018-0370-z

**Published:** 2018-03-12

**Authors:** Jinho Park, Du-Gyeong Han, Ji-Hyoung Ryu, Jeong-Byoung Chae, Joon-Seok Chae, Do-Hyeon Yu, Bae-Keun Park, Hyeon-Cheol Kim, Kyoung-Seong Choi

**Affiliations:** 1 College of Veterinary Medicine, Chonbuk National University, Iksan, 54596 Republic of Korea; 2 Department of Animal Science and Biotechnology, College of Ecology and Environmental Science, Kyungpook National University, Sangju, 37224 Republic of Korea; 3 Laboratory of Veterinary Internal Medicine, BK21 PLUS Program for Creative Veterinary Science Research, Research Institute for Veterinary Science and College of Veterinary Medicine, Seoul National University, Seoul, 08826 Republic of Korea; 4 Institute of Animal Medicine, College of Veterinary Medicine, Gyeongsang National University, Jinju, 52825 Republic of Korea; 5 College of Veterinary Medicine, Chungnam National University, Daejeon, 34134 Republic of Korea; 6 College of Veterinary Medicine, Kangwon National University, Chuncheon, 24341 Republic of Korea

**Keywords:** *Anaplasma bovis*, Grazing, Holstein cattle, Ticks

## Abstract

Anaplasmosis is a tick-borne infectious disease that affects both human and animal health. This study was performed to characterize and investigate the prevalence of infection with *Anaplasma bovis* in Holstein cattle originating from two regions in the Republic of Korea (ROK). Blood samples (n = 151; 80 from Namwon and 71 from Jeju Island) were analyzed by polymerase chain reaction, and the prevalence of *A. bovis* infection was compared before and after grazing. In Namwon, *A. bovis* infection was not detected, while in the Jeju Island, *A. bovis* infection was detected in three of 13 animals after grazing. Phylogenetic analysis revealed that the *A. bovis* isolates had homology (97.1–99.7%) with a Korean spotted deer (*Cervus nippon*) isolate and *Haemaphysalis longicornis* tick isolates identified in the ROK. *A. bovis* infection has not previously been diagnosed in cattle in the ROK. This study shows that *A. bovis* infection in the Jeju Island is closely related to grazing.

## Findings

The climate of the Korean Peninsula is rapidly becoming subtropical, and warmer temperatures have already resulted in accelerated parasitic development and an extreme rise in vector populations [1]. These climatic changes have a widespread impact on the ecosystems. Temporal and spatial changes in temperature, precipitation, and humidity that occur under different climatic conditions affect the biology and ecology of vectors and intermediate hosts, and may increase the risk of infection transmission [2]. Tick distribution is also closely linked with climate, and there is growing concern that the prevalence of tick-borne diseases, such as theileriosis and anaplasmosis, may be increasing in the Republic of Korea (ROK) [3–6].

Anaplasmosis is a tick-transmitted disease that affects dogs, cats, horses, cattle, sheep, goats, and wild ruminants. The *Anaplasma* genus comprises six species showing differences in host cell tropism. *A. centrale*, *A. marginale*, and *A. ovis* are erythrocytic, while *A. bovis*, *A. phagocytophilum*, and *A. platys* infect monocytes, neutrophils, and platelets, respectively [7]. Bovine anaplasmosis is caused by *A. bovis*, *A. centrale*, *A. marginale*, and *A. phagocytophilum. A. marginale* is widely distributed in tropical and subtropical regions throughout the world. It causes a mild to severe hemolytic disease in cattle and wild ruminants, and is particularly highly pathogenic in cattle up to 2 years old [8]. The infection is characterized by persistent fever, lethargy, icterus, weight loss, abortion, reduced milk production, and death in more than 50% of untreated animals [8]. *A. centrale* is a less pathogenic species compared to *A. marginale* and causes mild symptoms in cattle and is considered a naturally attenuated subspecies [9]. *A. phagocytophilum* is known to infect humans and animals, and causes tick-borne fever being characterized by fever, respiratory signs, leukopenia, abortion, and sudden decrease in milk production [10, 11]. *A. bovis* causes fever, anemia, drowsiness, convulsions, weight loss, and enlargement of lymph nodes in cattle [12]. This infection has been found in China and Japan [13–15], but recently, *A. bovis* was also detected in Korean spotted deer (*Cervus nippon*) [16], Korean water deer (*Hydropotes inermis argyropus*) [17], and *Haemaphysalis longicornis* ticks in the ROK [18, 19]. However, information regarding *A. bovis* infection in cattle is not available in the ROK. Therefore, the aim of the present study was to investigate *A. bovis* infection in cattle before and after grazing and to characterize the evolutionary relationships of obtained *A. bovis* isolates.

**Fig. 1:**
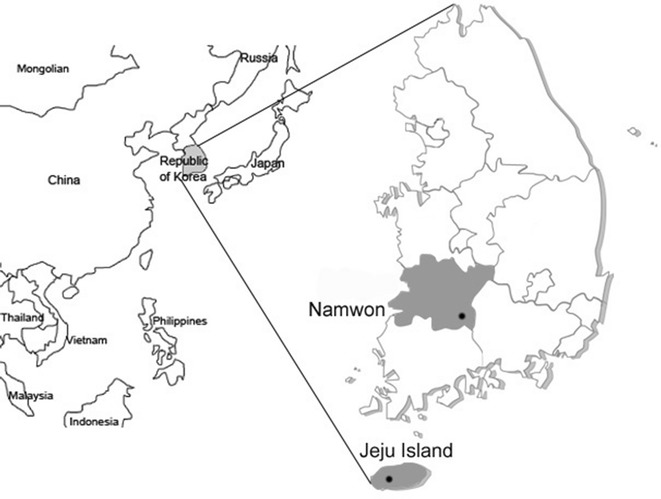
Map of the Republic of Korea. Dots indicate the location of the region of Namwon and the Jeju Island where blood samples were collected

Jugular vein EDTA stabilized blood samples (Vacutainer^®^ tubes, Beckton Dickinson, Franklin Lakes, NJ, USA) were taken from 151 Holstein cattle in the ROK, consisting of 80 samples from one herd in the Namwon region and 71 samples from a herd on the Jeju Island (Fig. 1). The samples were taken twice from April to August 2016. Cattle raised at both farms were grazed on grass from the middle of May to the end of November. The samples were analyzed for erythrocyte numbers, hemoglobin, hematocrit, and white blood cell counts using the VetScan HM5 Hematology System (Abaxis, Union, CA, USA).

Genomic DNA was extracted from blood samples using the DNeasy Blood kit (Qiagen, Hilden, Germany) according to the manufacturer’s instructions. A first round of polymerase chain reaction (PCR) was performed to amplify the 16S rRNA gene shared by all *Anaplasma* spp. (F, 5ʹ-TACCTCTGTGTTGTAGCTAACGC-3ʹ; R, 5ʹ-CTTGCGACATTGCAACCTATTGT-3ʹ). In a second round of PCR to identify individual *Anaplasma* spp., the following primers were used: AB1f/AB1r for *A. bovis* (F, 5′-CTCGTAGCTTGC TATGAGAAC-3′; R, 5′-TCTCCCGGACTCCAGTCTG-3′), *msp4* for *A. centrale* (F, 5′-CATGGGGCATGAATCTGTG-3′; R, 5′-AATTGGTTGCAGTGAGCGC-3′), and *msp4* for *A. marginale* (F, 5′-CATCTCCCATGAGTCACGAAGTGGC-3′; R, 5′-GCTGAACAG GAATCTTGCTCC-3′). PCR was performed under the cycling conditions: 98 °C for 5 min, followed by 35 cycles of 10 s at 98 °C, annealing at 58 °C for 30 s for 16S rRNA gene [3], 55 °C for 1 min for *A. bovis* [20], 53 °C for 30 s for *A. centrale*, and 53 °C for 30 s for *A. marginale* [21], 72 °C for 1 min, and final extension at 72 °C for 5 min. Distilled water was used as negative control for each PCR. The expected sizes of the 16S rRNA gene, *A. bovis*, *A. centrale*, and *A. marginale* were 429, 551, 395, and 252 bp, respectively. PCR products were visualized under UV light after 1.5% agarose gel electrophoresis and ethidium bromide staining.

The amplicons were purified using the Accupower Gel Extraction kit (Bioneer, Daejeon, ROK) and cloned into the pGEM^®^-T Easy vector (Promega, Madison, WI, USA), which was directly sequenced (Bioneer). Sequences were analyzed using the BioEdit version 7.2.5 sequence alignment software. A phylogenetic tree was constructed using the neighbor-joining method in MEGA 6.0 software [22] and bootstrapping with 1000 replicates. The two representative sequences obtained in this study were deposited in the GenBank database under accession numbers MF197897 and MF197898.

*Anaplasma bovis* was not detected in cattle from Namwon region, while three of 71 animals (4.2%) from Jeju Island tested positive (Table 1). No samples were positive for either *A. centrale* or *A. marginale*. None of the *A. bovis*-positive cattle showed hematological signs of infection, such as anemia and leukocytosis. *A. bovis* infection in cattle from Jeju Island was observed only after the animals had been on pasture consisted with having been exposed to ticks. This is the first study to report *A. bovis* infection in cattle in the ROK. The observed difference between the regions may be due to differences in climate. Unlike the Namwon region, Jeju Island has a subtropical climate with seasonal variations in precipitation, humidity, and temperature, which are more suitable for the reproduction and activity of ticks. Several studies have reported that the prevalence of *Anaplasma* spp. differs among climatic zones and is associated with suitability of tick habitats and animal management methods [13, 23].

**Table 1:** Comparison of *Anaplasma* infections before and after grazing in Namwon and Jeju Island

Region	Namwon		Jeju Island	
Date of sample collection	April 27, 2016	July 1, 2016	May 16, 2016	August 4, 2016
Grazing type/no. of samples	Housing (n = 40)	Grazing (n = 40)	Housing (n = 58)	Grazing (n = 13)
*A. bovis*	0	0	0	3
*A. centrale*	0	0	0	0
*A. marginale*	0	0	0	0

**Fig. 2:**
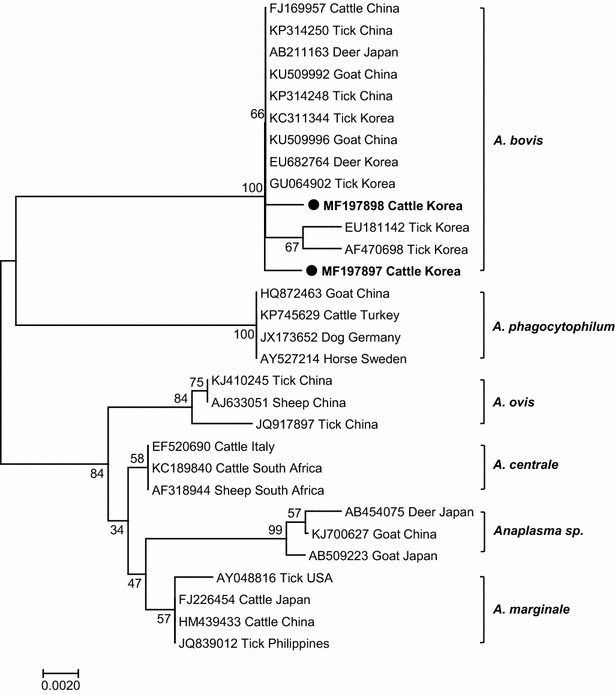
Phylogenetic analysis using the partial 16S rRNA gene (521 bp) sequences of isolates from Holstein cattle and representative Anaplasmataceae species. An unrooted phylogenetic tree was constructed with bootstrap values obtained by 1000 replicates using MEGA 6.0 software and the neighbor-joining method. The sequences found in this study are shown in bold

To detect *A. bovis* DNA in the blood, we first performed PCR using primers for the 16S rRNA gene shared by all *Anaplasma* spp. To identify *A. bovis*-infected cattle, PCR products were then amplified using *A. bovis*-specific primers (Table 1). Of the three *A. bovis* gene amplicons, two high-quality sequences were obtained (MF197897 and MF197898), which showed 98.1% homology. Phylogenetic analysis of the partial 16S rRNA gene was performed by aligning the obtained *A. bovis* sequences with selected *Anaplasma* spp. sequences found in GenBank. The MF197897 and MF197898 sequences were closely related to *A. bovis* and were distinct from *A. centrale*, *A. marginale*, *A. ovis*, *A. phagocytophilum*, and unspecified *Anaplasma* sp. included in GenBank (Fig. 2). The Korean cattle isolates had 99.7% homology to sequences from *A. bovis* strains originating from a Korean spotted deer (EU682764) and *H. longicornis* ticks (GU064902 and KC311344), respectively. They were also 97.1% homologous to sequences of *A. bovis* isolated from *H. longicornis* ticks (EU181142) from the Jeju Island and *H. longicornis* ticks (AF470698) collected from a different province (Gyeonggi) in the ROK (Fig. 2).

Knowledge on the epidemiology of *A. bovis* infection in cattle in the ROK is limited. *A. bovis* has been detected in *H. longicornis* ticks [18, 19, 24], the most common tick species in the ROK. This tick species may play an important role in the transmission of *A. bovis* infection in the ROK. Infection with *A. centrale* and *A. marginale*, the most common pathogens causing bovine anaplasmosis, were not found. This may be related to the absence of their vectors in the ROK. *Rhipicephalus simus* and *Dermacentor variabilis* are considered as tick vectors for *A. centrale* in Africa and *A. marginale* in the USA, respectively [23, 25]; however, in the ROK, these tick species have not been found. Although the clinical significance of *A. bovis* infection was not evaluated in the present study, extensive epidemiological studies on domestic animals are needed to clarify the pathogenicity and pathogenesis of *A. bovis* infection.

## Abbreviations

*A. bovis*: *Anaplasma bovis*
*A. centrale*: *Anaplasma centrale*
*A. marginale*: *Anaplasma marginale*
*H. longicornis*: *Haemaphysalis longicornis*
PCR: polymerase chain reaction
ROK: Republic of Korea
